# Improvement of ZrC/Zr Coating on the Interface Combination and Physical Properties of Diamond-Copper Composites Fabricated by Spark Plasma Sintering

**DOI:** 10.3390/ma12030475

**Published:** 2019-02-04

**Authors:** Yanpeng Pan, Xinbo He, Shubin Ren, Mao Wu, Xuanhui Qu

**Affiliations:** Institute for Advanced Materials and Technology, University of Science and Technology Beijing, Beijing 100083, China; pypxiwang@163.com (Y.P.); sbren@ustb.edu.cn (S.R.); wumao@ustb.edu.cn (M.W.); quxh@ustb.edu.cn (X.Q.)

**Keywords:** diamond-copper, composites, coating, sintering, physical properties

## Abstract

In this study, diamond-copper composites were prepared with ZrC/Zr-coated diamond powders by spark plasma sintering. The magnetron sputtering technique was employed to coat the diamond particles with a zirconium layer. After heat treatment, most of the zirconium reacted with the surface of diamond and was transformed into zirconium carbide. The remaining zirconium on the zirconium carbide surface formed the outer layer. Owing to the method used to produce the ZrC/Zr-coated diamond in this study, the maximum thermal conductivity (TC) of 609 W·m^−1^·K^−1^ was obtained for 60 vol. % diamond-copper composites and the corresponding coefficient of thermal expansion (CTE) reached as low as 6.75 × 10^−6^ K^−1^. The bending strength of 40 vol. % ZrC/Zr-coated diamond-copper composites reached 255.95 MPa. The thermal and mechanical properties of ZrC/Zr-coated diamond-copper composites were substantially superior to those of uncoated diamond particles. Excellent properties can be attributed to the strengthening of the interfacial combination and the decrease in the interfacial thermal resistance due to the improvement associated with the ZrC/Zr coating. Theoretical analysis was also proposed to compare the thermal conductivities and CTE of diamond-copper composites fabricated with these two kinds of diamond powders.

## 1. Introduction

Conventional electronic packaging materials such as W/Mo-Cu [1], SiC_p_-Cu/Al [2,3] all display limited thermal conductivities (TCs). With the continual improvement of the chip’s integration level and complexity of the circuit board in the field of microelectronic packaging, it is imperative to develop the next generation of heat sink materials with high-temperature TC [4,5]. Diamonds with regular morphology and low nitrogen content generally have extraordinarily high TCs and low CTEs, which can be used to produce high-thermal-conductivity electronic packaging materials with a copper matrix. As the next generation of heat sink materials, diamond-copper composites have recently received an increasing amount of attention with respect to their use in the field of electronic packaging [6,7,8]. 

The major challenges in preparing diamond-copper composites are the weak combination and high thermal resistance at the interface which limits the comprehensive properties. Without introducing elements to optimize wettability at the interface, the TC of the composite has been significantly lower than that of pure copper. To address these issues, two primary methods were used to improve the interface wettability. First, matrix alloying was employed, whereby active elements are added into the copper matrix to establish chemical interactions with diamond. Forming a thin nano- or micrometer-size carbide layer and adding minor amounts of active elements especially Zr [9,10], B [11], Ti [12,13], and Cr [14,15,16], can significantly optimize the interfacial combination. Apparently, the alloying elements inevitably diffuse into the copper matrix, which greatly reduces the TC of diamond-copper composites. Another method is to coat diamond with B [17,18], Ti [19,20], Cr [21,22], Mo [23,24,25,26], or W [27,28,29,30] by vacuum micro-deposition, molten salt bath, or magnetron sputter deposition. As W and Mo are not soluble in copper, the bonding forces with copper are less than that of Ti [31], Cr [22], and Zr. Zirconium is normally added as bulk into the copper matrix by the high temperature-high pressure [9] or gas pressure infiltration method [10]. The highest TC of 930 W·m^−1^·K^−1^ was obtained with the addition of zirconium into copper matrix by infiltration. The bonding improvement of the interface depends on the amount of zirconium added and the sintering temperature. Small quantities of zirconium can form a thin ZrC layer with diamond, and the remaining elemental residue resides in the copper, which can reduce the thermal diffusivity of the matrix. In this study, magnetron sputtering was used to coat the zirconium directly on the diamond surface. The zirconium transformed into zirconium carbide with heat treatment, and in this way, the formation of a ZrC layer is easier and more efficient compared to the addition of zirconium.

The preparation method is also a crucial factor for high performance diamond-copper composites. In the past few years, diamond-copper composites have commonly been produced by PM and metal infiltration techniques. One of the powder metallurgy methods, spark plasma sintering, is thought to be a convenient and effective way to produce diamond-copper composites [32,33,34]. Using a pulse current to heat the composite powder, the sintering time can be limited to 5–20 min. In this study, spark plasma sintering was used to prepare ZrC/Zr-coated diamond-copper composites for the first time. After heat treatment, zirconium reacted with the diamond surface, and the inner ZrC layer thereon formed firm chemical bonding. With rapid sintering by spark plasma sintering (SPS), the outer zirconium layer partly diffused into the copper matrix, so the copper matrix and zirconium layer had good interfacial adhesion. With this well-designed coating layer on the surface of diamond particles, both the coating layer with diamond and copper matrix effectively combined. Thus, the excellent interface was beneficial to both thermophysical and mechanical properties. To obtain high-volume-fraction diamond-copper composites using the powder metallurgy method, mixed sizes of diamond particles with a certain ratio were used in the reinforcement phases. With the small size particles filling in the gaps of larger ones, the density of the composite can be effectively increased.

## 2. Experimental Procedures

### 2.1. Preparation of Composite

Electrolytic copper powders (Xinrongyuan Metal Powders Co., Ltd., Beijing, China) with an average diameter of 75 μm and purity of 99.9% were used as the matrix. The reinforcements were synthetic MBD8-grade diamond powders with the average sizes of 100 and 40 μm (Polaris Diamond Powder Co., Ltd., Beijing, China). TC of diamond is excellent when the nitrogen content is effectively controlled. A small amount of zirconium was plated onto a diamond surface by adjusting the sputtering parameters. The surfaces of diamond particles were physically cleaned using ultrasound to remove the impurities before magnetron sputtering. To maintain purity of the coating layer, a target material with 99.9 wt. % zirconium was selected. Ar was selected as the sputtering gas and the zirconium target was bombed by Ar^+^ under an intense magnetic field. The furnace was pumped until it reached low vacuum conditions of 1 × 10^−3^ Pa. The surface of diamond particles was entirely clad in zirconium after sputtering. Heat treatment at 700 °C for 30 min was conducted in a vacuum atmosphere. The pure zirconium layer gradually transformed into a zirconium carbide layer from the inside out. To ensure composite densification, diamond particles of two different sizes (100 μm/40 μm = 3:1) were used. The hybrid diamond powders were mixed with copper powders to prepare 40–60 vol. % diamond-copper blended powders. Using argon gas atmosphere protection, it is possible to effectively avoid copper powders from being oxidized. To avoid the zirconium carbide coating layer from being worn off, a few ceramic balls were added to the powder blending equipment. The prepared powders were wrapped in a graphite die and then synthesized using the SPS equipment. The size of the synthesized specimen was Φ 30 mm × 4 mm. The pressure was maintained at 40 MPa throughout synthesis. The composites with different diamond volume fractions were heated to 900 °C and held for 20 min. The heating rate was 100 °C/min. The synthesized disk-shaped samples were cut into designated shapes using a laser cutter for material performance testing.

### 2.2. Property Testing

The polished and fracture surfaces of composites were investigated by JSM-6510A SEM (Scanning Electron Microscope, JEOL Ltd., Tokyo, Japan). The interfacial atomic composition was detected by attached EDS (Energy Dispersive Spectroscopy, JEOL Ltd., Tokyo, Japan). XRD (X-ray diffraction, Siemens D5000, Siemens, Munich, Germany) analysis was conducted to identify the phases of zirconium coating layer and diamond-copper composites. The actual density was measured using the Archimedes’ principle. To avoid water permeation, the sample surface was coated with a thin film of Vaseline. The thermal diffusivities and specific heat were measured with LFA 427 Nanoflash (Netzsch, Selb, Germany). TC of the composite was deduced by the product of thermal diffusivity, specific heat, and density. The CTE curves with increasing temperature were obtained by Netzsch DIL 402C dilatometer (Netzsch, Selb, Germany) in an argon atmosphere. The samples were continuously heated from 25 °C to 300 °C with a heating rate of 5 °C/min.

## 3. Results and Discussion

### 3.1. Characteristics of the Zirconium Carbide Coating

**Figure 1:**
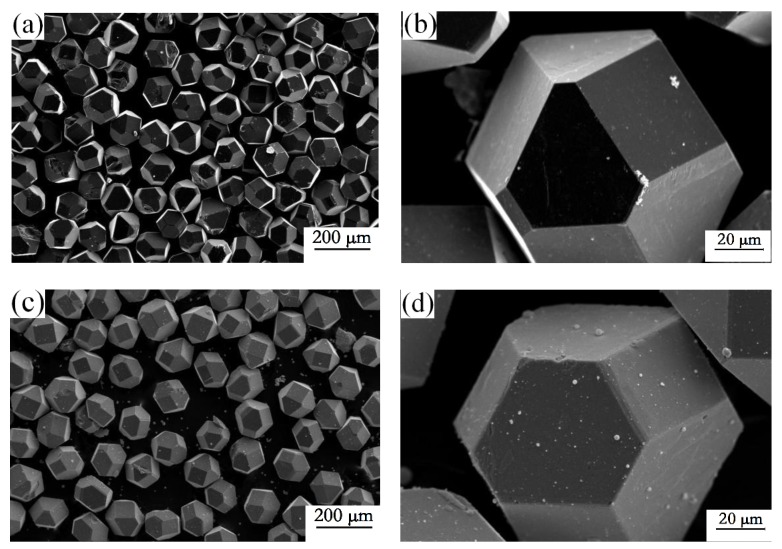
Micro-morphology of (**a**,**b**) uncoated and (**c**,**d**) ZrC/Zr-coated diamond particles.

Figure 1a,b show the morphologies of purchased diamond particles. The raw diamond was hexagonal or octahedral in shape. After magnetron sputtering and heat treatment, the diamond surfaces were evenly coated with zirconium carbide, as shown in Figure 1c,d. As diamond surfaces were physically coated with zirconium and a slight carbonization reaction occurred on the diamond surface during heat treatment, the diamond particles maintained good surface morphology. There were no coating defects or flaws.

**Figure 2:**
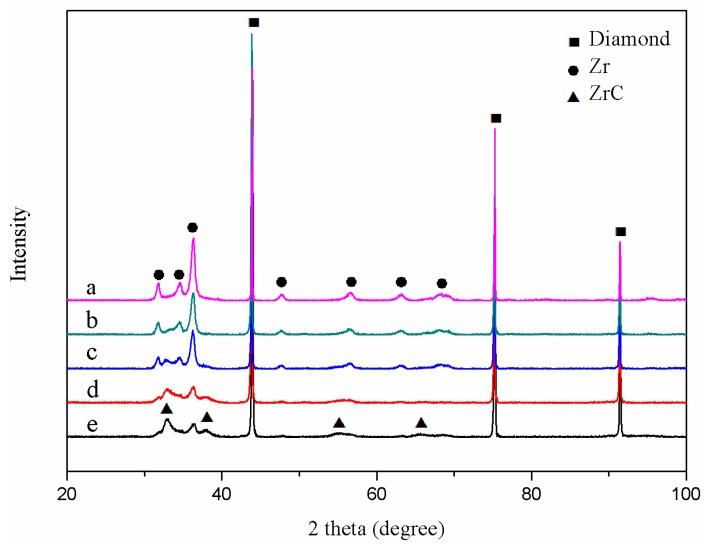
XRD of ZrC/Zr-coated diamond with different heat treatment time: (**a**) 0 min; (**b**) 5 min; (**c**) 10 min; (**d**) 20 min and (**e**) 30 min.

X-ray diffraction patterns of ZrC/Zr-coated diamond are illustrated in Figure 2. As shown in Figure 2a, only pure zirconium peaks were detected after magnetron sputtering. During heat treatment, zirconium gradually reacted with the diamond surface and transformed into zirconium carbide. The peak intensity of zirconium decreased, and that of zirconium carbide was gradually detected, as shown in Figure 2b–d. XRD patterns were indicative of the decrease of zirconium thickness and the transformation of the pure zirconium layer to zirconium carbide. At the end of the carbonization reaction, the inner ZrC layer formed, and the thin outer zirconium layer remained. A ZrC/Zr dual-layer coating structure formed, and the major ZrC peaks with weak Zr peaks were confirmed, as shown in Figure 2e. 

**Figure 3:**
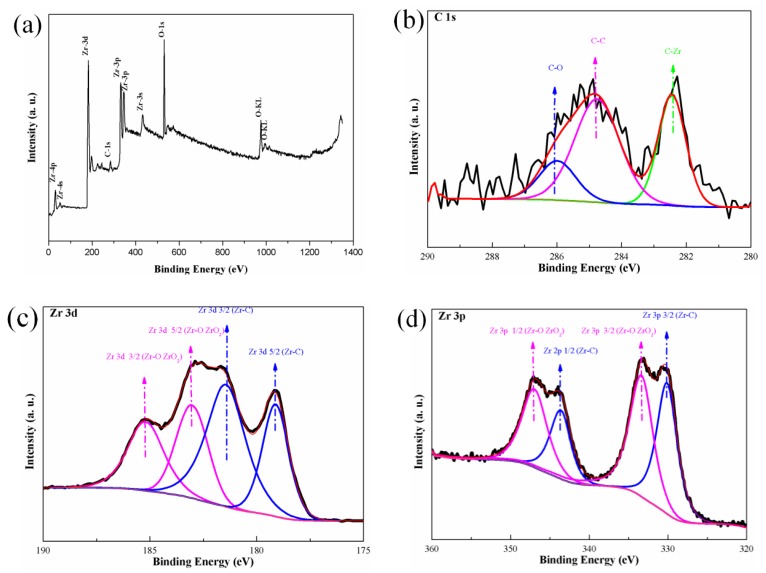
XPS patterns of ZrC/Zr-coated diamond particles: (**a**) full-scanning spectra; (**b**) narrow-scanning spectra of C1s; (**c**) narrow-scanning spectra of Zr3d; and (**d**) narrow-scanning spectra of Zr3p.

Phase state of zirconium-carbide-coated diamond was further investigated by XPS (X-ray Photoelectron Spectroscopy, Thermo Scientific Escalab 250Xi, Thermo Fisher Scientific Ltd., Waltham, MA, USA) analysis (Figure 3). By combining the XPS and XRD methods, the compositions and phases were accurately obtained. As shown in Figure 3b, the C1s peak indicates the binding energies of C atoms (282.44 and 284.76 eV), representing Zr–C and C–C bonds respectively. Zr mainly existed as Zr–C and Zr–O bonds. After heat treatment, the zirconium coating layer reacted with the surface of the diamond, and a ZrC layer formed. The Zr–C bond was detected both in the narrow scanning of Zr3d (179.12 and 181.44 eV) and Zr3p (330.13 and 343.6 eV). Zirconium was easily oxidized and deposited as a zirconia (CZ) residue on diamond particles. Zr–O bond (183.03 and 185.17 eV in Zr3d, and 333.36 and 346.96 eV in Zr3p) came primarily from ZrO_2_. XPS analysis was applied without surface etching, and the zirconia existed only as a thin film on the outer zirconium surface. Pure zirconium has a strong affinity for oxygen, and it tended to form a thin zirconia film on the surface. Surface oxidation mainly occurred because of the unintended introduction of oxygen. During the storage period, the outer zirconium layer of the diamond particles came into direct contact with air. Isolating zirconium from the source of oxygen can effectively avoid contamination of the diamond surface.

### 3.2. Microstructure of the Diamond-Copper Composites

**Figure 4:**
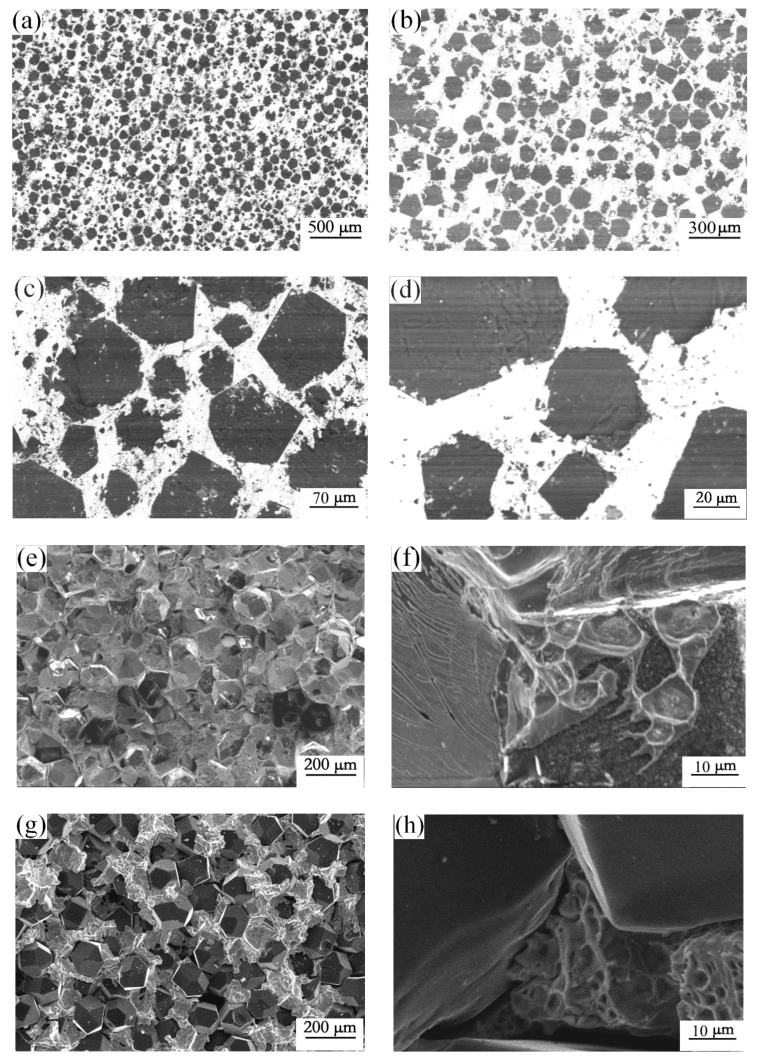
Micro-morphology of diamond-copper composites: (**a**–**d**) the polished surface of the composites; (**e**,**f**) fracture surface of ZrC/Zr-coated diamond-copper composites; (**g**,**h**) fracture surface of uncoated diamond-copper composites.

Figure 4 shows the microstructure of diamond-copper composites. As can be seen in Figure 4a,b, the diamond particles were mixed with two sizes of diamond particles with a specific weight ratio of 3:1, as illustrated in Figure 4c,d. Small-sized diamond particles can fill in the gaps among larger-sized diamond particles, whereby the density of the composite can be effectively improved. Figure 4e,f show the composite microstructure of the fracture surface prepared with ZrC/Zr-coated diamond. The coated diamond displayed a notable improvement of the interfacial bonding with a copper matrix. The ZrC/Zr layer exhibited a good interfacial bonding, and no cracks were observed, as shown in Figure 4f. In contrast, the uncoated diamond particles were exposed on the fracture surface, being poorly combined with the copper matrix (Figure 4g). The fracture surface of uncoated diamond/Cu composites showed obvious gaps between diamond and the copper matrix (Figure 4h).

**Figure 5:**
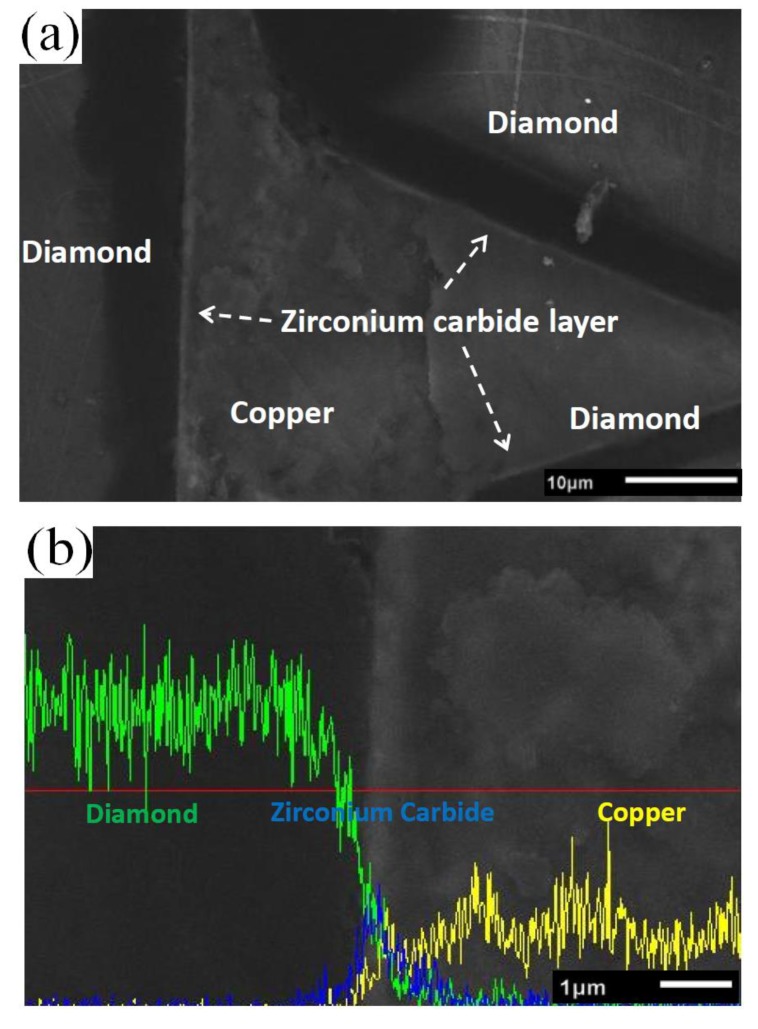
(**a**) The interface ZrC layer of diamond-copper composites; (**b**) EDS interface line-scanning in ZrC/Zr-coated diamond-copper composites.

A low solubility of zirconium in copper matrix can ensure the TC of the copper matrix. On the basis of the sintering characteristic of SPS, the rapid shaping process effectively holds the original form of the diamond coating layer. Irrespective of the small amount of zirconium on the diamond surface diffusing into the copper matrix, the ZrC/Zr layer was homogeneous at the interface, as shown in Figure 5a. Three separate zones existed among the interface; namely, the matrix, the interface layer, and the reinforcement particles. The sintering temperature was not high, and the sintering time was limited. The outer zirconium layer did not completely diffuse into the matrix. The zirconium layer near the copper became ambiguous, as shown in Figure 5b. EDS interface line-scanning was adopted to analyze the elemental distribution at the interface. A sharp increase in intensity of zirconium was obtained with the elemental line scan from the copper matrix to the diamond particle, indicating that the ZrC/Zr layer exhibited relatively strong bonding at the interface.

### 3.3. Thermal Conductivity of Diamond-Copper Composites

The TCs of diamond-copper composites prepared with uncoated and ZrC/Zr-coated diamond particles were measured. The TC of ZrC/Zr-coated diamond-copper composites showed a dramatic improvement compared to that of the uncoated ones. With an increase in diamond content, the TC reached a maximum of 609 W·m^−1^·K^−1^. 

The contribution of the coating layer to the interfacial thermal conductivity was then analyzed using the H-J model. With the H-J model, a series of factors such as reinforcement size, volume fraction, and interface combination status were considered to estimate TC. The H-J model is a widely accepted theory to describe TC of a composite and can be expressed with the following equation [17,21,22]:

(1) $$\lambda_{c}=\lambda_{m}\left[\frac{2\left(\frac{\lambda_{d}}{\lambda_{m}}-\frac{R\lambda_{d}}{a}-1\right)V_{d}+\frac{\lambda_{d}}{\lambda_{m}}+\frac{2R\lambda_{d}}{a}+2}{\left(1-\frac{\lambda_{d}}{\lambda_{m}}+\frac{R\lambda_{d}}{a}\right)V_{d}+\frac{\lambda_{d}}{\lambda_{m}}+\frac{2R\lambda_{d}}{a}+2}\right]$$

where *λ* is TC, R is thermal resistance, *V* is the reinforced phase content, and *a* is the size of reinforced phase. Meanwhile, *d*, *m*, and *c* represent diamond, copper and composite in this study.

Another theoretical model to evaluate the TC of composites is the DEM scheme shown below [10,31]:

(2) $$\left(1{-V}_{d}\right){\left(\frac{\lambda_{c}}{\lambda_{m}}\right)}^{1/3}=\frac{\frac{\lambda_{d}}{\lambda_{m}\left(1+\frac{R\lambda_{d}}{a}\right)}-\frac{\lambda_{c}}{\lambda_{m}}}{\frac{\lambda_{d}}{\lambda_{m}\left(1+\frac{R\lambda_{d}}{a}\right)}-1}$$

The interfacial thermal resistance *R* is a crucial factor in the theoretical calculations of Equations (1) and (2). The sintering temperature and time of the spark plasma sintering process cannot guarantee the complete diffusion of the zirconium into matrix. Both the Zr and ZrC layers exist at the interface. On the basis of the structure of the ZrC/Zr coating layer, the interfacial thermal resistance consists of these terms. The first terms are the thermal resistances at the diamond-ZrC interface, ZrC-Zr interface, and Zr-copper interface. The second terms are the intrinsic interfacial thermal resistances of the ZrC layer and Zr layer. Therefore, the total interface thermal resistance can be expressed as *R* = *R*_diamond/ZrC_ + *R*_ZrC_ + *R*_ZrC/Zr_ + *R*_Zr_ + *R*_Zr/Cu_. The interface between the ZrC and Zr layers gradually formed because of the carbonization reaction. Zirconium partly diffused into the copper matrix and formed a blurring interface where the zirconium element emerged with a gradient distribution. For these reasons, the thickness of the ZrC and Zr layers cannot be precisely ascertained. Thus, by ignoring the elements’ distribution states at the interface, the theoretical interfacial thermal resistance *R* was directly calculated, with the phonon velocity parameters of diamond and copper being integrated into the theoretical model. 

The value of *R* can be estimated using the AMM theory, a simple Debye model which defines phonon as the primary form to transfer energy by passing through the interface. The thermal resistance *R* is shown as [17,22]:

(3) $$R=\frac{2v_{d}}{C_{m}\rho_{d}}\cdot {\left(\frac{\rho_{m}v_{m}+\rho_{d}v_{d}}{\rho_{m}v_{m}^{2}}\right)}^{2}$$

where *C* is the specific heat, *ρ* is the density, and *v* is the phonon velocity. Meanwhile, *m* and *d* represent copper and diamond in this study. Particularly, *v* can be expressed as:

(4) $$v={\left(\frac{3}{v_{t}^{2}+2v_{l}^{2}}\right)}^{1/2}v_{t}v_{l}$$

where *t* refers to the transverse direction and *l* refers to the longitudinal direction. The parameters of the copper matrix and diamond reinforcement were substituted into Equations (3) and (4), and the value of thermal resistance *R* (2.08 × 10^−8^ m^2^·K·W^−1^) was obtained.

**Figure 6:**
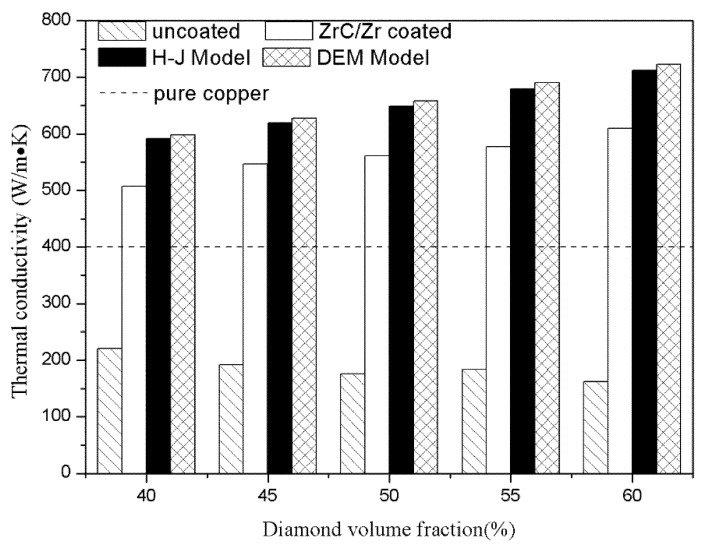
TCs of uncoated and ZrC/Zr-coated diamond-copper composites in comparison with theoretical values.

The thermal resistance was calculated using the Debye model, which can be substituted into the two models mentioned above to obtain the theoretical TC of the composites (Figure 6). Based on the calculation results, the measured values of the composites fabricated with ZrC/Zr-coated diamond particles were consistent with the predicted ones of the H-J and DEM models, and the TC values of uncoated diamond-copper composites were much lower than that of pure copper.

### 3.4. Coefficient of Thermal Expansion of the Diamond-Copper Composites

**Figure 7:**
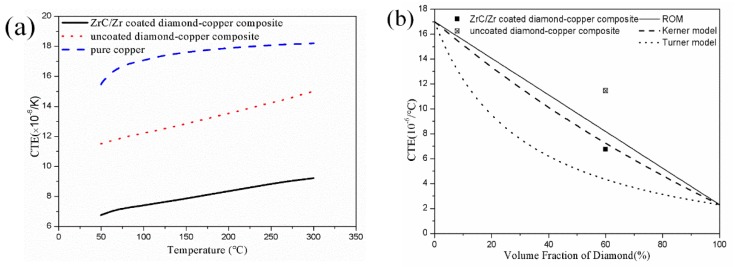
(**a**) Experimental CTE values of uncoated and ZrC/Zr-coated 60 vol. % diamond-copper composites; (**b**) Comparison of the CTEs between the experimental results and theoretical models.

The match of CTE between substrate plate and microelectronic devices is also crucial for electronic packaging. The measured values of the composites with increasing temperature are shown in Figure 7a. The ROM, Turner, and Kerner models are three original models for predicting composite CTE [10,12,14].

The ROM model is expressed as:

(5) $$\alpha_{c}=\alpha_{m}V_{m}+\alpha_{d}V_{d}$$

The Turner model assumes that shear deformation can be neglected with the homogeneously strain in the composite. The CTE of the composite is shown as:

(6) $$\alpha_{c}=\frac{\alpha_{m}V_{m}K_{m}+\alpha_{d}V_{d}K_{d}}{V_{m}K_{m}+V_{d}K_{d}}$$

where *α* is CTE, *V* is the reinforced phase content and *K* is the bulk modulus. By taking shear deformation into consideration, the Kerner model can be expressed as:

(7) $$\alpha_{c}=\alpha_{d}V_{d}+\alpha_{m}V_{m}+(\alpha_{d}-\alpha_{m})V_{d}V_{m}\times \frac{K_{d}-K_{m}}{V_{d}K_{d}+V_{m}K_{m}+(3K_{d}K_{m}/4G_{m})}$$

where *G_m_* represent shear modulus of diamond-copper composite. As can be seen in Figure 7a, the CTE values of ZrC/Zr-coated diamond-copper composite are much lower than that of pure copper and uncoated ones. At 50 °C, the CTE value of the 60 vol. % ZrC/Zr-coated diamond/Cu composite reached 6.75 × 10^−6^ K^−1^. The measured value well agreed with the value calculated by the Kerner model rather than the ROM or Turner model, as shown in Figure 7b. This can account for both normal and shear stress existing in the composites as a result of the temperature increase and the Kerner model taking both factors into account. By contrast, the CTE value of the composite fabricated with uncoated diamond reached 11.45 × 10^−6^ K^−1^, which is much higher than that of the coated ones. When the metal substrate and diamond particles were heated, the weak bonding between phases failed to limit expansion of the copper matrix on account of their different expansion coefficients. For this reason, the CTE of uncoated diamond/Cu composite was much higher. Meanwhile, the size of ZrC layer ranging from 0.3–0.5 μm effectively improved the bonding strength between diamond particles and the copper matrix, decreasing CTE considerably. 

### 3.5. Mechanical Characterizations of the Diamond-Copper Composites

**Figure 8:**
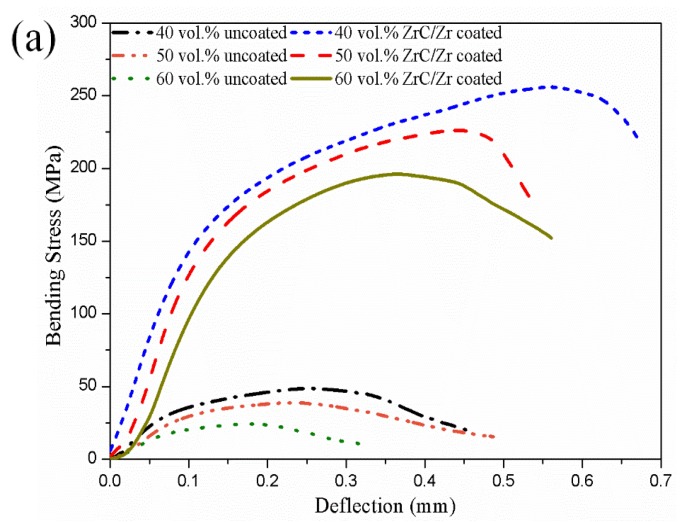

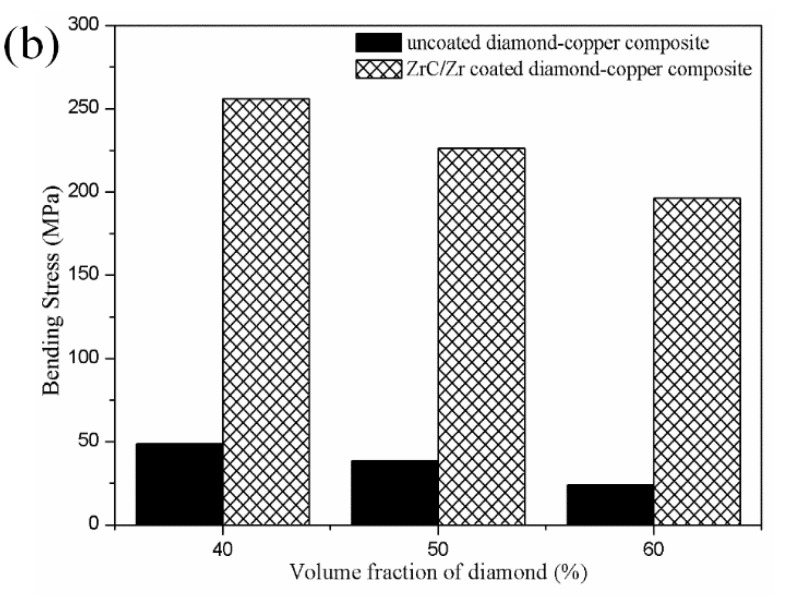
(**a**) Bending stress-deflection curves of diamond-copper composites; (**b**) bending strength of uncoated and ZrC/Zr-coated diamond-copper composites.

The mechanical properties of diamond-copper composites can also reflect the interfacial bonding between diamond particles and the copper matrix. Figure 8a shows the bending stress-deflection curves of diamond-copper composites. With the improvement of interfacial combination by the ZrC/Zr layer, bonding strength was augmented significantly. The inner ZrC layer formed strong chemical bonding with diamond particles after heat treatment. The outer Zr layer partly diffused into the copper matrix, then enhanced metallurgical bonding. With the well-designed interface structure, the ZrC/Zr layer limited the shear deformation between diamond and copper under shear load. The bending strength of 40 vol. % ZrC/Zr-coated diamond/Cu composite reached 255.95 MPa. Contrarily, the bending stress of uncoated diamond/Cu composite with the same volume fraction reached only 48.62 MPa (Figure 8b).

## 4. Conclusions

By magnetron sputtering, diamond surface was deposited with zirconium. After heat treatment in a vacuum environment, zirconium reacted with the diamond surface and transformed into zirconium carbide. Diamond-copper composites were then produced with this ZrC/Zr-coated diamond of mixed particle sizes by SPS technique. The composites produced with uncoated diamond particles displayed low thermal conductivities. As diamond is non-wetting with copper, gaps exist at the interface, which can reduce the bonding strength. The addition of ZrC/Zr onto the diamond surface using the magnetron sputtering technique can effectively enhance the interfacial bonding. The highest TC reached 609 W·m^−1^·K^−1^, with a corresponding CTE of 6.75 × 10^−6^ K^−1^. In addition, the bending strength of 40 vol. % ZrC/Zr-coated diamond-copper composite reached 255.95 MPa. The excellent properties of these coated diamond-copper composites are a promising candidate to meet the demand for a variety of electronic packaging applications.
